# Native Predators Do Not Influence Invasion Success of Pacific Lionfish on Caribbean Reefs

**DOI:** 10.1371/journal.pone.0068259

**Published:** 2013-07-11

**Authors:** Serena Hackerott, Abel Valdivia, Stephanie J. Green, Isabelle M. Côté, Courtney E. Cox, Lad Akins, Craig A. Layman, William F. Precht, John F. Bruno

**Affiliations:** 1 Department of Biology, The University of North Carolina at Chapel Hill, Chapel Hill, North Carolina, United States of America; 2 Department of Biological Sciences, Simon Fraser University, Burnaby, BC, Canada; 3 REEF, Key Largo, Florida, United States of America; 4 Marine Sciences Program, Florida International University, North Miami, Florida, United States of America; 5 NOAA – Florida Keys National Marine Sanctuary, Key Largo, Florida, United States of America; McGill University, Canada

## Abstract

Biotic resistance, the process by which new colonists are excluded from a community by predation from and/or competition with resident species, can prevent or limit species invasions. We examined whether biotic resistance by native predators on Caribbean coral reefs has influenced the invasion success of red lionfishes (*Pterois volitans* and *Pterois miles*), piscivores from the Indo-Pacific. Specifically, we surveyed the abundance (density and biomass) of lionfish and native predatory fishes that could interact with lionfish (either through predation or competition) on 71 reefs in three biogeographic regions of the Caribbean. We recorded protection status of the reefs, and abiotic variables including depth, habitat type, and wind/wave exposure at each site. We found no relationship between the density or biomass of lionfish and that of native predators. However, lionfish densities were significantly lower on windward sites, potentially because of habitat preferences, and in marine protected areas, most likely because of ongoing removal efforts by reserve managers. Our results suggest that interactions with native predators do not influence the colonization or post-establishment population density of invasive lionfish on Caribbean reefs.

## Introduction

Indo-Pacific lionfishes (*Pterois volitans* and *Pterois miles*, hereafter termed “lionfish”) are the first exotic marine fish to successfully invade and become established across the greater Caribbean region [1]. The invasion has the potential to significantly alter marine ecosystems through competition with native predators and predation on reef fishes and invertebrates. Some of these anticipated effects are already evident. For example, Green et al. [2] reported a 65% decline in native fish biomass over just two years on heavily invaded reefs of New Providence, Bahamas. On experimental reefs, a single lionfish was shown to reduce the recruitment and richness of native fishes [3], and have significantly greater impacts than that of a comparable native mesopredator (the coney grouper, *Cephalopholis fulva*) [4]. Lionfish are predicted to have substantial cascading impacts on coral reef food-webs [5] and benthic community structure [6].

The recent invasion of Caribbean coral reefs by lionfish has alarmed reef managers, who are racing to identify effective mitigation strategies. The best management strategies, in terms of regulating lionfish spread and abundance, are likely to be derived from an understanding of the processes underpinning lionfish invasion success. Introduced species may successfully invade novel areas because the biotic agents regulating their populations (e.g., competitors, predators) are rare or absent in the new range [7] – the enemy release hypothesis [7]. Thus, recipient communities with high fish biodiversity and abundance may be more likely to resist establishment of invasive species than communities that are less species-rich – the diversity-invasibility hypothesis [8].

In the case of introduced lionfish, healthy populations of native predatory fishes could “resist” lionfish colonization, either indirectly through competition for habitat and prey resources, or more directly through predation – the biotic resistance hypothesis [9], [10]. Lionfish have been found in the stomach contents of large groupers [11], consumed by moral eels [12], and sharks in some areas have been taught to consume dead lionfish [13]. However, it is unclear how often predation on lionfish occurs naturally. In an apparent example of biotic resistance, Mumby et al. [14] found that lionfish biomass was lower within the Exuma Cays Land and Sea Park, which has relatively high predator biomass, suggesting that interactions with predators, specifically large groupers, may reduce the post-establishment abundance of lionfish.

The purpose of our study was to test the generality of those findings, by examining the relationship between native predator and lionfish abundance in the Caribbean on a large, regional, scale. Over three years, we surveyed 71 reefs in three regions of the Caribbean. We hypothesized that if biotic resistance plays a role in limiting the lionfish invasion, lionfish abundance (density and biomass) should be negatively related to the abundance of native predatory fish. Because reefs varied not only in native predator abundance, but also in depth, habitat structure, and time since lionfish invasion, we also explored potential relationships between lionfish abundance and these abiotic conditions.

## Methods

### Ethics Statement

No endangered or protected species were involved in this field study. No vertebrates were collected in this study; surveys were through visual census only.

### Study Sites and Fish Abundance

**Figure 1:**
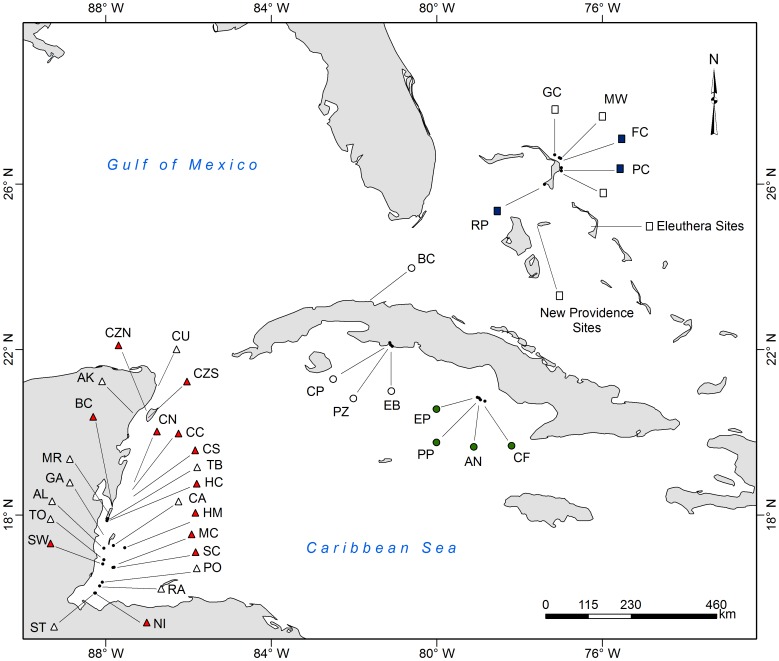
Location of survey sites. Location of surveys sites. For site abbreviations, surveys dates and coordinates refer to Table S1.

We surveyed 71 coral reefs (3–15 m deep) across spur-and-grove, slope, and patch reef habitats in three regions of the Caribbean: The Bahamas, Cuba, and the Mesoamerican Barrier Reef in Belize and Mexico, from 2009 to 2012 (Table S1, Fig. 1). Sites were selected to cover a wide range of reef fish abundance. Appropriate permission was obtained for each survey location where a permit was required (Text S1). Due to the range of habitat types included and the large scale of our analysis, we modified survey methods (time and area) to suite each reef type. At each reef site, we conducted underwater visual census to survey fish abundance using belt transects (modified from AGRRA v5.0 [15]), except at Eleuthera Island, Bahamas, where we performed roving survey dives, which were more appropriate for the patch reef habitats [2]. At each continuous reef site, we randomly placed six to eight belt transects parallel to the spur-and-groove formations or along the reef slope habitat following constant isobaths. All surveys for lionfish included a thorough search of the benthos for cryptic fish. Reef fish species were identified, counted, and sizes were estimated in 10 cm intervals. Fish of <40 cm total length (TL) were counted in six to eight 30×2 m belt transects, while fish of >40 cm TL and lionfish were counted in six to eight 50×10 m belt transects. When the spurs of the spur-and-groove habitat were shorter than 50 m, we counted lionfish and large fish along the longest transect possible (i.e., 20, 25 or 30 m by 10 m). At each site, the larger and wider transect contained the smaller transect, except in New Providence, Bahamas, where the larger and smaller transects did not overlap. At Eleuthera, we estimated the area of each patch reef surveyed to calculate fish densities.

We first calculated the abundance: density (standardized to individual 100 m^−2^) and biomass (standardized to g 100 m^−2^), of the native predatory fish species observed during each survey that could potentially prey upon and/or compete with lionfish and then calculated mean values per site (see a list of the sampled species in Table S2). Fish biomass for each species was calculated through the allometric length-weight conversion formula, ***W = aTLb***, where *W* is the weight of each individual fish in grams, *TL* is the total length recorded for each fish in cm, and the parameters *a* and *b* are species-specific [16]. We used the mid-point of the 10 cm interval to calculate biomass. The variables *a* and *b* were obtained from FishBase [16], selecting the values from areas that were geographically closest to our study region. When these variables were not available, we used the values of congeneric species of similar size and morphology.

In addition to considering the abundance of all native predators combined (“total predators”), predators were also divided in two size categories: “small predators” (<40 cm TL) and “large predators” (>40 cm TL). We hypothesized that “small predators” may affect lionfish through competition for resources or through predation on juvenile lionfish, while “large predators” might control lionfish through predation on both juveniles and adults. We also examined the relationship between the abundance of lionfish and particular grouper species. We hypothesized that relatively large grouper species, such as Nassau (*Epinephelus striatus*), tiger (*Mycteroperca tigris*), and black (*Mycteroperca bonaci*) grouper, were the most likely to prey on lionfish [11], [14], and each species was analyzed separately. Similarly, three mesopredator species, including coney (*Cephalopholis fulva*), graysby (*Cephalopholis cruentata*), and red hind (*Epinephelus guttatus*), were identified, based on similarity of foraging habitat and prey items, as most likely to compete with lionfish, although they may also prey on juvenile lionfish [5]. Each species was analyzed separately.

### Abiotic Covariates

To account for potential variability in lionfish abundance unrelated to interspecific interactions (i.e., predation and competition), we considered six potential geographic and abiotic covariates. Three variables were continuous: latitude (in decimal degrees), mean survey depth (in meters) for each site, and time (in years) between the first sighting of lionfish in each of the study regions and the survey year [1]. Three covariates were categorical: habitat type (spur-and-groove, slope, and patch reef), protection level (protected = in a “no-take” marine reserve (n = 17) or not = unprotected (n = 55)), and wind/wave exposure (windward or leeward). We tested for correlation among our continuous predictor variables by constructing a Spearman correlation matrix, which indicated that that *Latitude* and *Time since invasion* were significantly correlated (r = 0.55, p<0.001). This is likely because the invasion started at higher latitude (Florida) and progressed to lower latitudes (Belize) [1]. To avoid the problems arising by modeling correlated variables together, we dropped *Latitude* from the analysis and used *Time since invasion* because the later might better reflect the impact on reef communities. We used the coordinates of each site to account for potential spatial auto-correlation of lionfish abundance among sites (detailed below).

### Models of Lionfish Density and Analysis

Since the data have a spatial component, we accounted for spatial autocorrelation (i.e. lack of independence among sample units) to decrease the chances of type I errors [17]. We used spline correlograms of the raw lionfish data as graphical representations of the correlation between sites at a range of lag distance, calculated from *Latitude* and *Longitude* [18]. This approach uses a bootstrap algorithm with 1000 permutations to build a confidence envelope around the entire covariance function [18]. The original lionfish abundance data was spatially auto-correlated. To account for the hierarchical structure of our data, e.g., transects nested within sites and sites nested within regions, and the potential for spatial autocorrelation, we used Generalized Linear Mixed Effects Modeling (GLMM). GLMM accounts for dependencies within hierarchical groups through the introduction of random effects [19], [20].

Lionfish counts at the transect-level were modeled as a function of the belt-transect or patch reef area, e.g., for Eleuthera. We modeled lionfish abundance as a function of the native predator abundance (density and biomass) and abiotic site variables using the Automatic Differentiation Model Builder (glmmADMB) package in R [15], which accounts for over-dispersed data with an excess of zeros [20]. We evaluated several models of lionfish abundance that included biotic and abiotic variables using the Akaike Information Criterion (AIC) as a relative comparable measure of goodness of fit among models. Based on the lowest AIC scores, our data were best described by a generalized linear mixed effect model [12]. The lionfish abundance data were negatively binomially distributed (as assessed visually and with a distribution fitting function in R) within sites, and approximately 58% of all transects or roving surveys had no lionfish. Thus we set the zero-inflation portion of the model as “true” to account for these two issues [21]. We used lionfish counts in each model as a response variable, and added the log of survey area per transect (i.e., belt transect areas) as an offset to account for the positive relationship between survey area and number of fishes observed, as well as to account for the negative binomial distribution. We used site as a random factor nested within region (Bahamas, Cuba, and Belize-Mexico) and the remaining variables were considered fixed. We standardized (centered and divided by standard deviation) the continuous abiotic covariates to aid model convergence. Thus, the number of lionfish *Y* on transect (or roving survey) *t*, was described as
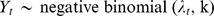
where *λ_t_* is the number of lionfish per transect, and *k* is the over-dispersion parameter. The density of lionfish at transect *i* (*µ_i_* ) was related to the number of lionfish per transect (*λ_t_*) and the survey area per transect (*A_t_*) as



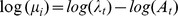



The complete model was set as
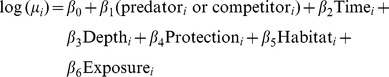
where *β*_0_ is the intercept and *β*_j_ is the regression parameter of the model that correspond to each covariate.

Lionfish biomass was positively and strongly correlated with lionfish density (Fig. S1, r = 0.95, p<0.01). We modeled both lionfish density and biomass as response variables; however, because the results were qualitatively identical, we only report the density results for simplicity. We used density and biomass of each predator group as covariates. We ran 10 different models; five with density and five using the biomass of the five biotic groups (total predators, large predators, small predators, total grouper, and a combination of specific species of interest [black+Nassau+tiger+coney+graysby+red hind]). All models were run with the same abiotic covariates. We did not detect multi-collinearity among abundance or biomass for the species of interest, thus we analyzed them in the same model.

Spline correlograms constructed from Pearson residuals of all of the GLMM models indicated that our mixed-effect modeling framework successfully accommodated spatial autocorrelation observed in the raw data (Fig S2.). We used Mantel tests [22] to check for overall autocorrelation between the Pearson residuals of each model and distance between sites (i.e, whether sites that are closer together were more similar), and found that correlation coefficients for all models were r <0.073 (p<0.0001). We performed the autocorrelation analyses in R version 2.14.2 [15] using the package *ncf* version 1.1.4 [23].

## Results and Discussion

Lionfish density in our study ranged from 0 at several sites in Belize, Cuba, and The Bahamas to ∼52 individuals 100 m^−2^ on a patch reef off Eleuthera Island, Bahamas, with an average of 4.4 individuals 100 m^−2^ (+/−0.5, SE). This range is comparable to lionfish densities found in New Providence, Bahamas [24], [25] and in North Carolina [26]. Total native predator biomass was significantly higher in the 17 marine reserves we sampled (Fig. S3) and ranged widely, from 4 g 100 m^−2^ on some spur-and-groove reefs off New Providence, Bahamas, to 51700 g 100 m^−2^ in slope habitats of Jardines de la Reina marine reserve, Cuba. The highest predatory fish biomass value of our sites (51700 g 100 m^−2^) is comparable to some isolated reefs of the central Pacific [27], [28]. A wide range of predator biomass has also been reported across the Caribbean [29]. This 1000-fold difference of total predator biomass among reefs is associated with human population density [30], [31] and likely due in part to variable fishing pressure [16], [31], as well as the loss of reef structural complexity related to coral mortality [31], [32].

**Figure 2:**
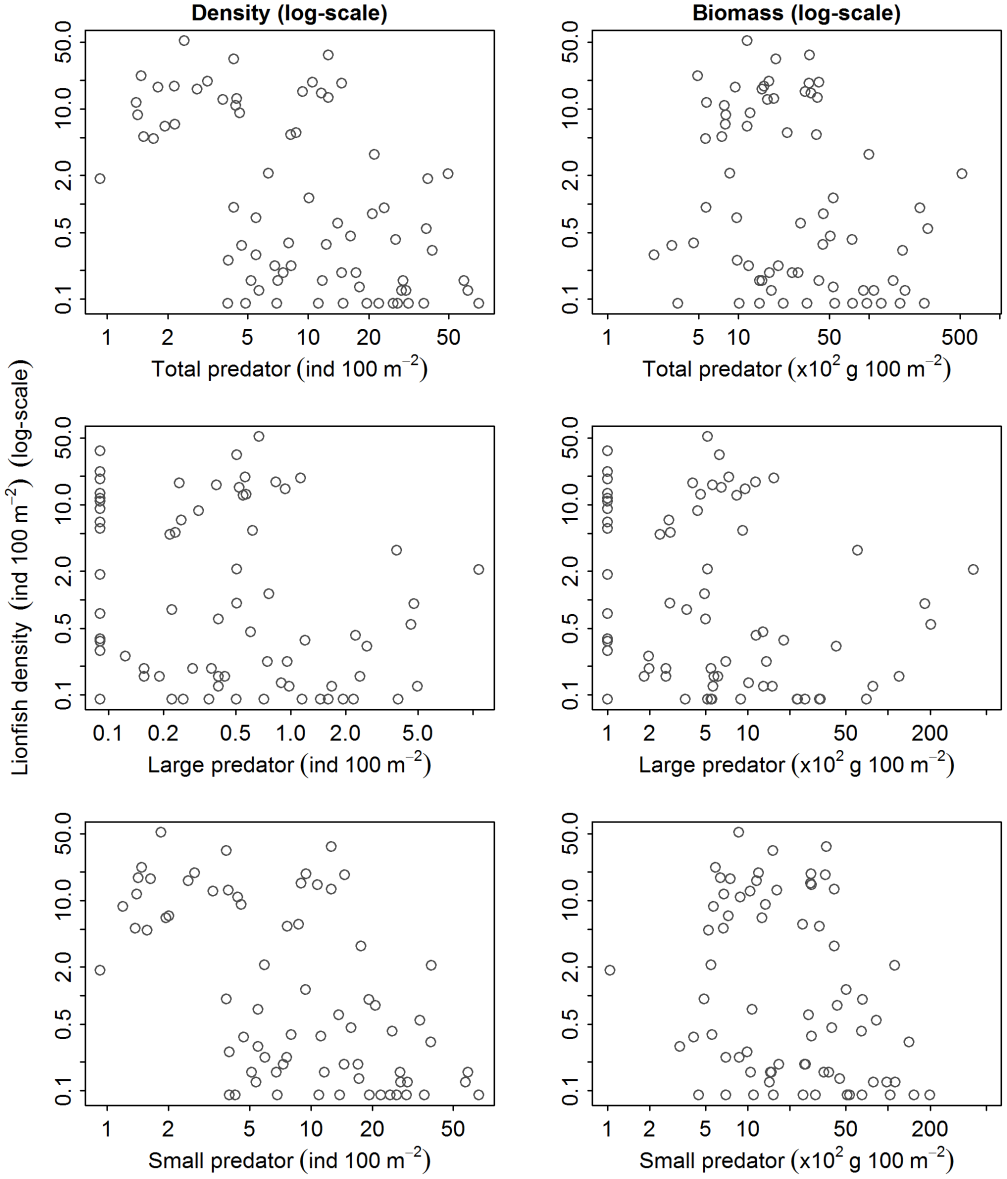
Relationship between lionfish density (ind 100 m^−2^) and abundance of native Caribbean predatory fishes (density [ind 100 m^−2^ ] and biomass [g 100 m^−2^]). Lionfish density versus “total predators”, “large predators”, and “small predators” density and biomass.

**Figure 3:**
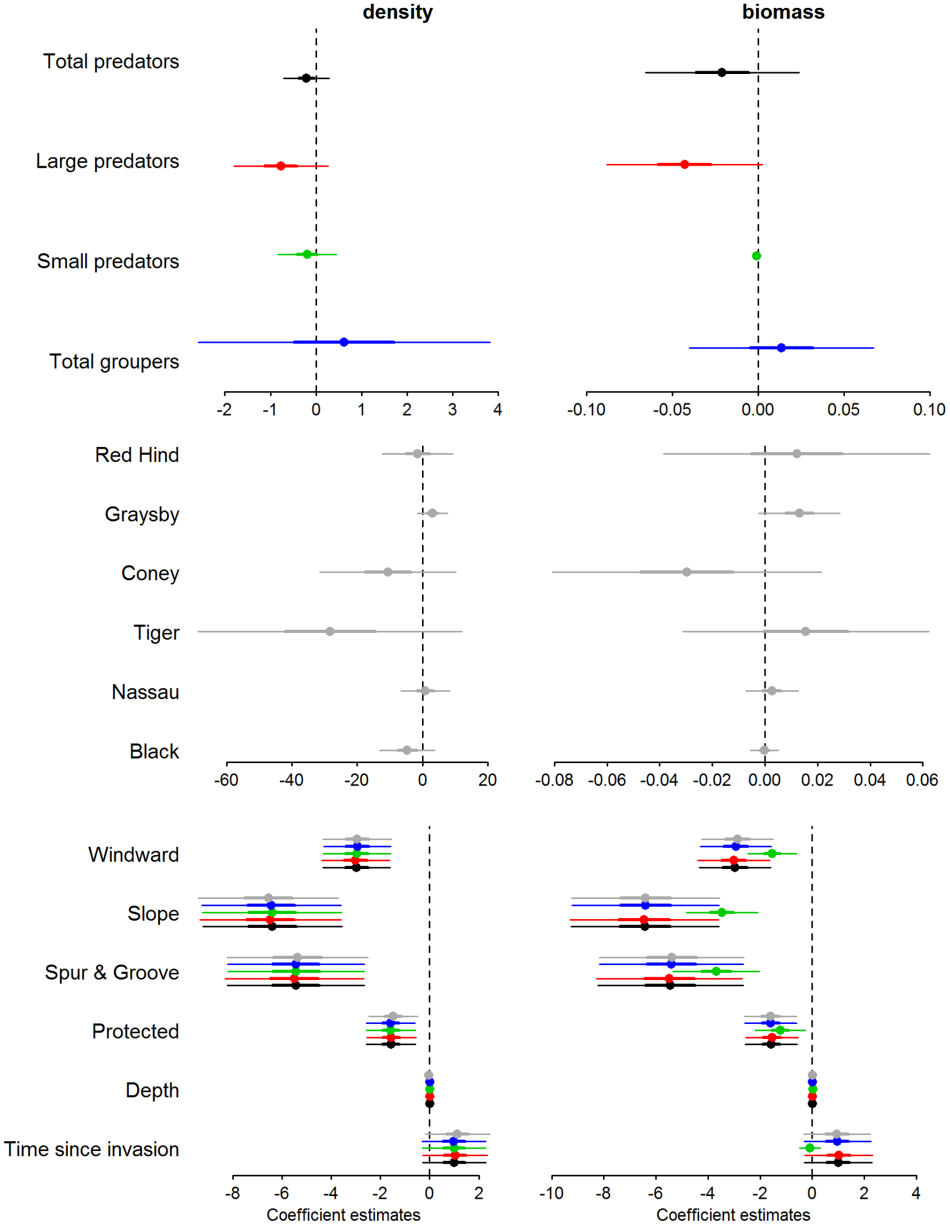
Coefficient estimates (mean, ±1 standard deviation and ±95% confidence interval) for each of the glmmADMB models. We ran ten models, five with density and five with biomass of biotic groups. Every model was run with the same abiotic factors. The biotic groups were: total predators, large predators, small predators, total grouper, and groupers by species (black+Nassau+tiger+coney+graysby+red hind). Each color represents a model for either density or biomass of biotic groups.

**Figure 4:**
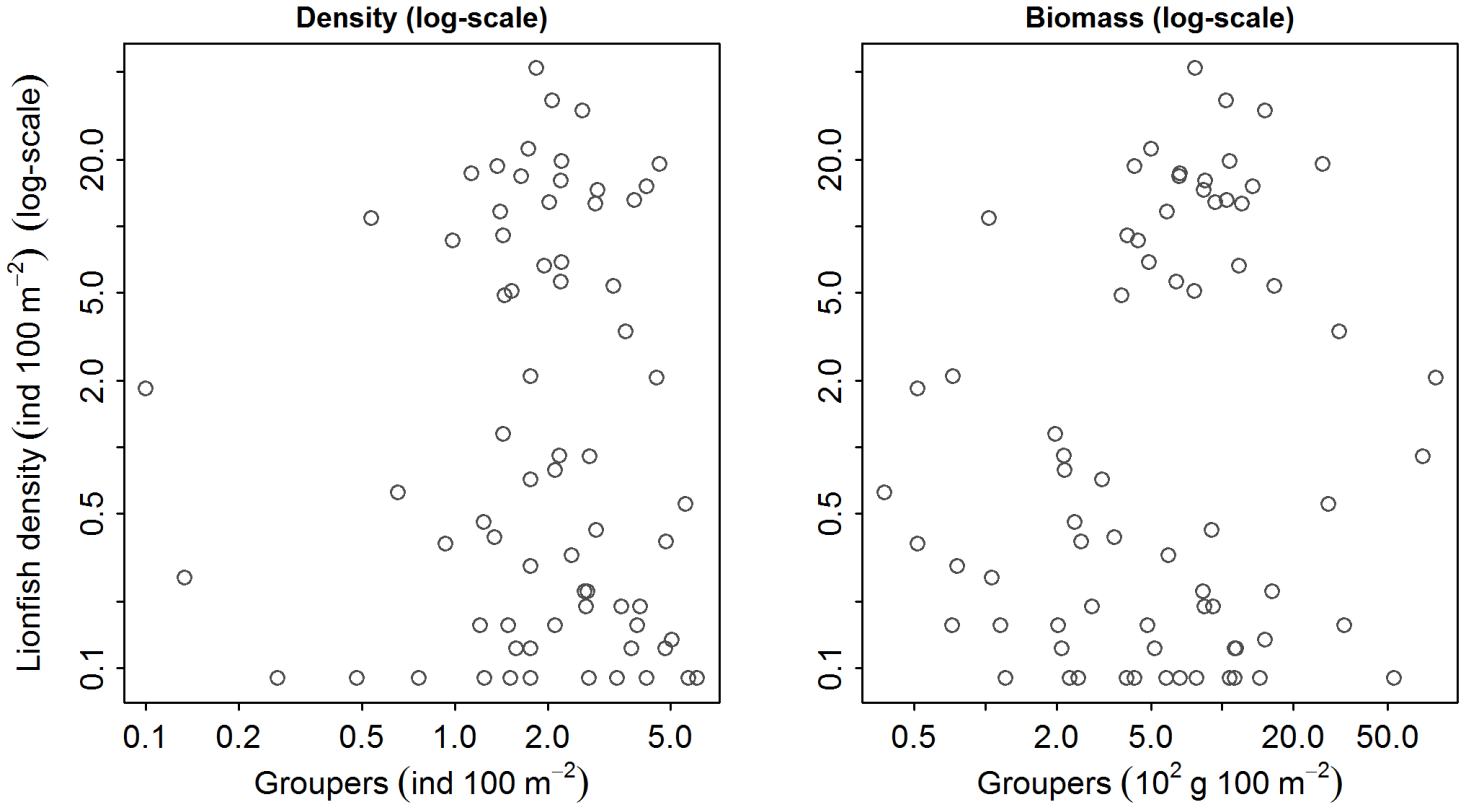
Relationship between lionfish density (ind 100 m^−2^) and total grouper abundance (density [ind m^−2^] and biomass [g m^−2^]). Lionfish density versus total grouper biomass and density.

We did not detect a significant relationship between lionfish density and any metrics of native predator abundance, which included both aggregative measures of abundance (i.e., biomass and density) of total predators, large predators, small predators (Fig. 2 and 3), or total grouper (Fig. 3 and 4). Likewise, we did not detect a significant relationship between lionfish density and the abundance of species that may occupy a similar ecological role to lionfish, including tiger grouper, Nassau grouper, black grouper, red hind, graysby, and coney (Fig. 3, 5–6). Our results suggest that interactions between native predators and lionfish on Caribbean reefs are not influencing lionfish densities. Lionfish were present at high densities on reefs with abundant, as well as depauperate, native predator assemblages. For example, in the Jardines de la Reina marine reserve in Cuba, where predator biomass was the highest observed (average ∼30000 g 100 m^−2^), lionfish were just as abundant as on the Mesoamerican Barrier Reef sites (1.5 individuals 100 m^−2^ in Jardines de la Reina and 1.6 individuals 100 m^−2^ on Mesoamerican Barrier Reef) that have far lower predator biomass (average ∼2200 g 100 m^−2^). Similarly, evidence from the Flower Garden Banks National Marine Sanctuary reveals an increasing population of lionfish despite high diversity and biomass of native predators [33].

**Figure 5:**
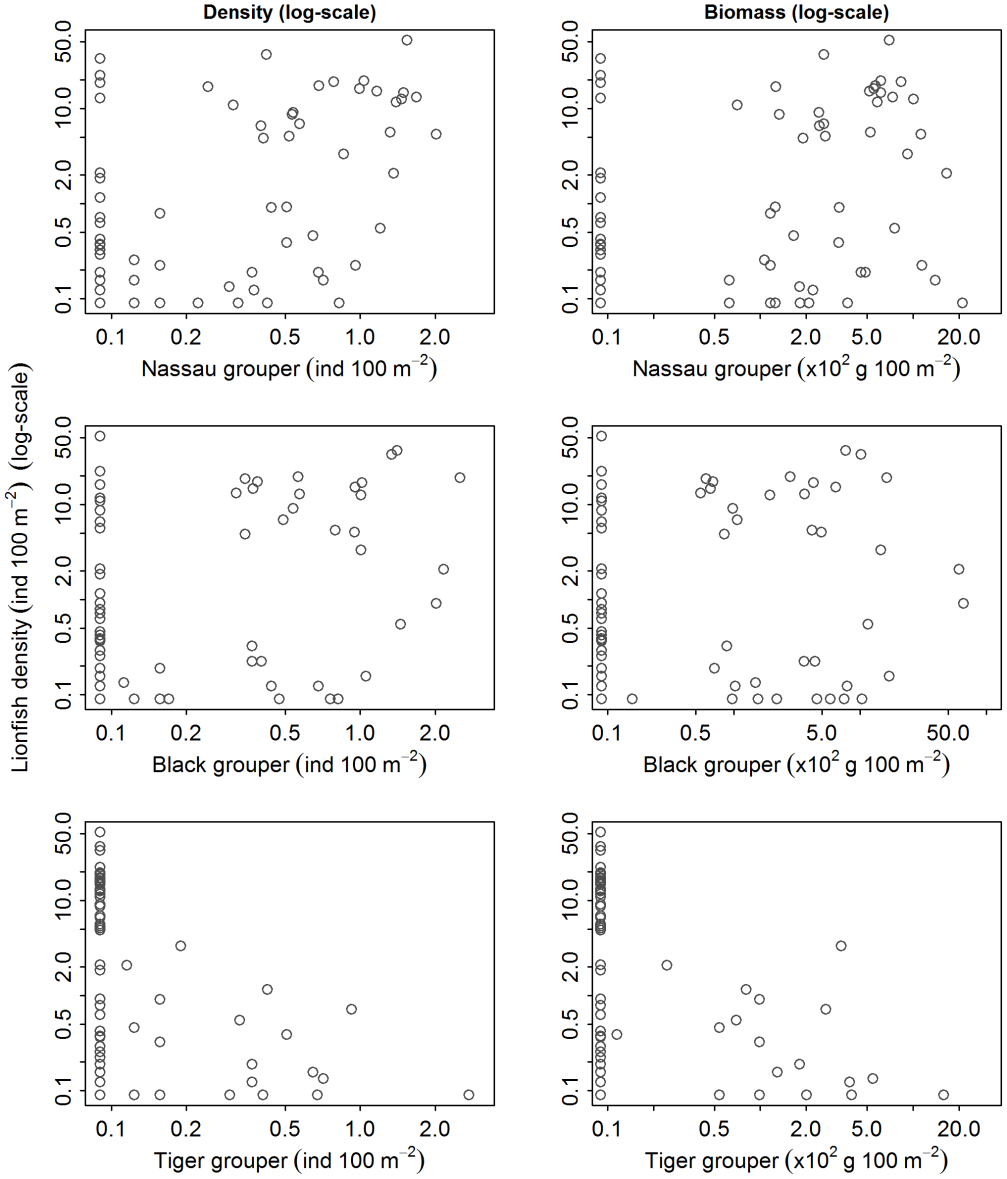
Relationship between lionfish density (ind 100 m^−2^) and abundance of large Caribbean groupers (density [ind 100 m^−2^] and biomass [g 100 m^−2^]). Lionfish density versus biomass and density of Nassau, black, and tiger grouper.

**Figure 6:**
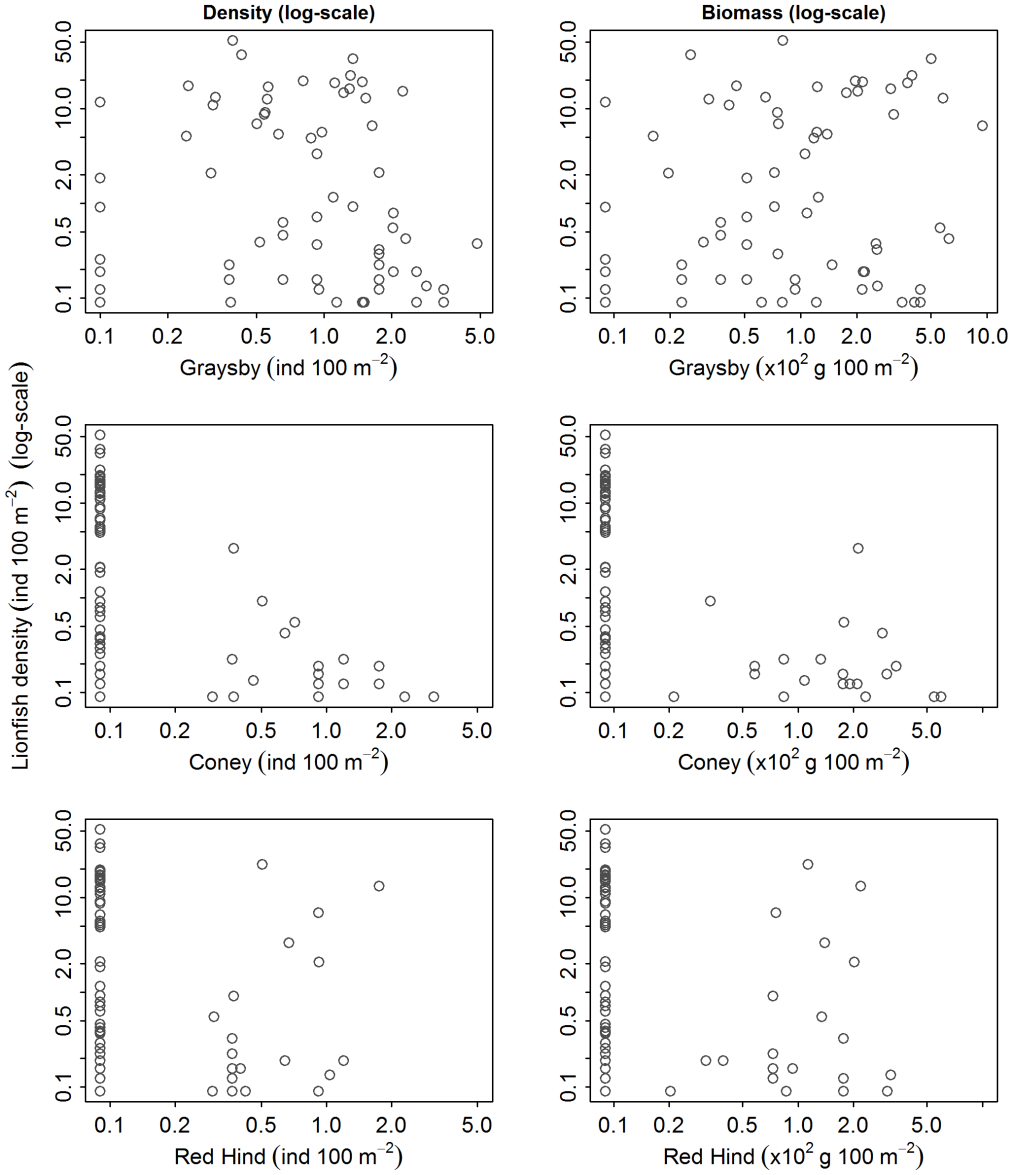
Relationship between lionfish density (ind 100 m^−2^) and abundance of potential native competitors (density [ind 100 m^−2^] and biomass [g 100 m^−2^]). Lionfish density versus biomass and density of coney, graysby, and red hind.

**Figure 7:**
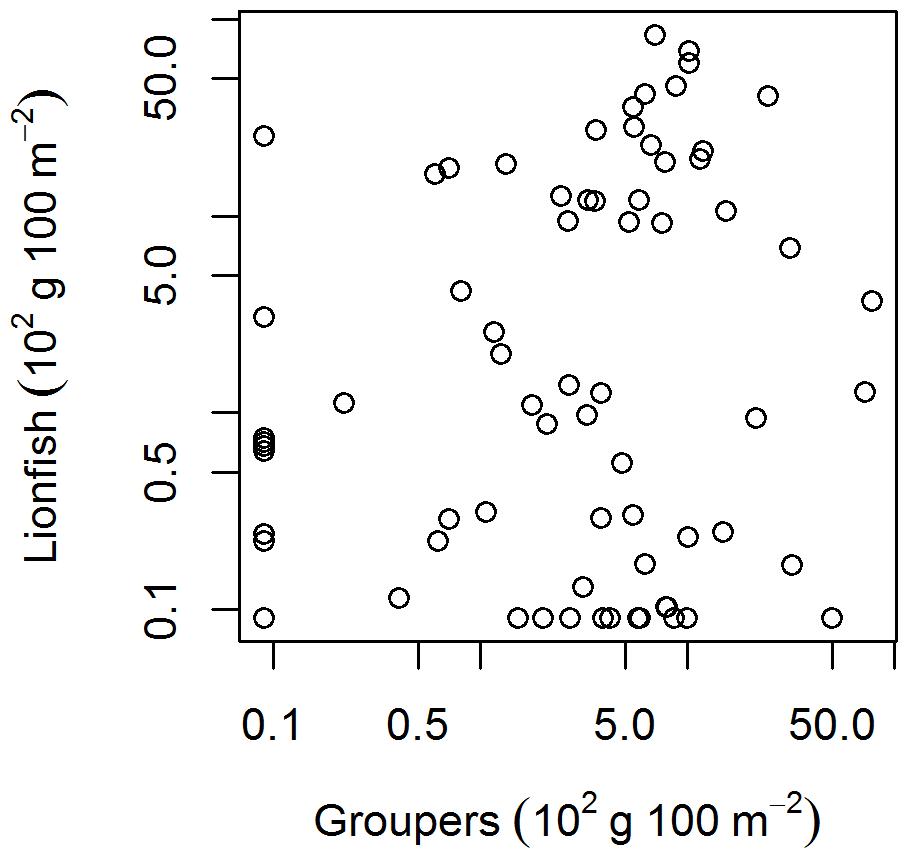
The relationship between the biomass of large grouper species and lionfish biomass. Note the log-log scale. Each point represents a site mean. GLMM analysis indicated lionfish and large grouper biomass were not significantly related. “Grouper” in this plot includes all “large grouper species” as defined by Mumby et al. [14], i.e., *Epinephelus striatus* (nassau grouper), *Mycteroperca tigris* (tiger grouper), *M. bonaci* (black grouper), *M. venenosa* (yellowfin grouper), and *M. interstitialis* (yellowmouth grouper).

Our results partly contradict those of Mumby et al. [11], who found an inverse relationship between lionfish and large grouper biomass within the Exuma Land and Sea Park (ELSP) and argued this was due to biotic resistance, through predation by large groupers. The biomass levels of “large grouper” in our study (0–7835 g 100 m^−2^, Fig. 7) exceeded the range reported by Mumby et al. [14] for the Exuma islands, where the maximum biomass of large grouper was ∼ 2500 g 100 m^−2^. Thus, the observed lack of effect of native predators on lionfish in our study was not due to limited variation in predator abundance.

Alternatively, predation can perhaps limit lionfish densities at the onset of an invasion but not once populations are well established. Lionfish abundance was quite low in the Exuma study; our observed regional mean and maximum for lionfish biomass (range 0–8000 g 100 m^−2^, mean = 781, SE = 84, n = 71 sites) were ∼10X and ∼100X greater (respectively) than the values reported for reefs around the Exuma islands [14]. This suggests that either the lionfish colonization was still at a very early stage when the Exuma reefs were surveyed or that low propagule pressure or some environmental characteristic is greatly limiting lionfish populations there, both in protected and unprotected sites. In fact, the observed reduction of lionfish biomass in the ELSP represents just ∼ 0.5% of our observed regional variance, suggesting a modest relative effect size.

A lack of association between the abundance of potential predators and that of older juvenile/adult lionfish could potentially arise for a number of reasons, most of which are not mutually exclusive. First, in many fish populations, the strongest population bottlenecks occur at the settlement stage or immediately after [34]. Thus if biotic resistance occurs, it would be most evident during earlier life stages. Second, many fish species show ontogenetic habitat shifts, with recruits and young juveniles being spatially segregated from adults. To our knowledge, however, such habitat separation does not occur in lionfish: very small juveniles and adults are commonly observed in close proximity (all authors, pers. obs.). Finally, it is possible that predator presence, density, biomass, or composition do not influence lionfish density or biomass, but rather some other metric of individual- or population-level fitness, such as hunting efficiency, fecundity, or larval export, all of which could influence the spread and population dynamics of this invasive predator.

Biotic resistance by predators does not appear to be a general phenomenon controlling lionfish. Lionfish densities may be lower in marine reserves; however, this effect is independent of predator abundance. Grouper biomass was confounded with site protection status in the ELSP study [11], i.e., all the sites with highest grouper biomass were within the reserve. It would therefore have been difficult to distinguish an effect of grouper per se, from an effect of protection status, including differences in habitat condition [35], [36] and potential culling of lionfish in the reserve. Our study was designed to overcome this limitation by sampling a large number of reefs, both within and out of reserves, and across a gradient of native predator biomass. Doing so enabled us to separate the role of these co-varying site characteristics in influencing lionfish density and biomass. Indeed, we found that there are fewer lionfish within reserves, but that predator and grouper biomass have no measurable effects on lionfish abundance. It is likely that lionfish densities are lower in marine reserves due to targeted removal. Lionfish are regularly culled from most Caribbean reserves by managers, dive operations, and tourists in efforts to preserve the integrity of the protected reefs. Research evaluating the efficacy of local removals as a tool in lionfish control is on-going [37], and some results are promising [38]–[40].

While the restoration of large reef predators is an essential conservation priority for the Greater Caribbean that would enhance a range of ecosystems services and greatly influence reef community dynamics, based on our results, it would not measurably mitigate the rapid spread of invasive lionfish. Active and direct management, perhaps in the form of sustained culling, appears to be essential to curbing local lionfish abundance and efforts to promote such activities should be encouraged.

## Supporting Information

Figure S1 Relationship between lionfish density and biomass estimates. Each point represents a transect mean. The Pearson’s product-moment correlation between lionfish biomass and lionfish density was 0.95, p<0.01.
(TIFF)

Figure S2 Spline correlograms, with 95% point wise bootstrap confidence intervals, of the Pearson residuals for each generalized liner mixed effects logistic regression model including all the explanatory variables fitted to the data.
(TIFF)

Figure S3 Total predator biomass on protected and unprotected Caribbean reefs. The biomass of native predatory fishes on 17 protected sites (no-take marine reserves) and on 55 unprotected or non-reserve reefs. Average predator biomass was significantly higher at sites inside marine reserves (135.4 g/m^2^) than in non-reserve sites (37.7 g/m^2^); t = −4.5933, p = 1.05e-05 (t-test). Boxplot shows the mean (black dot), median (black line) values.
(TIFF)

Table S1 Survey locations across the Caribbean Study sites, site codes, regions, and protection level. Habitat type, S&G: Spur and Grove; Patch: Patch Reef. Protection level, NTZ: No-take zone; MPA: marine protected area; GUA: general used area. Permit, Yes: Permit Obtained (Permits for The Bahamas, Belize, and Mexico covered all sites); Not Req.: Permit was not required and therefore not obtained (For Cuba, only protected sites required a permit).
(PDF)

Table S2 Reef fish predator species used in the study. Taxonomic information, food guild and trophic groups of the predator species used in the analysis. Guild and trophic information was obtained from Fish-Base [16].
(PDF)

Text S1 Field survey permit information.
(DOCX)
