# Supplementary figures and images for: Native Predators Do Not Influence Invasion Success of Pacific Lionfish on Caribbean Reefs
Source: PLoS One. 2013 Jul 11;8(7):e68259. doi: 10.1371/journal.pone.0068259 (PMC3708960; doi:10.1371/journal.pone.0068259)

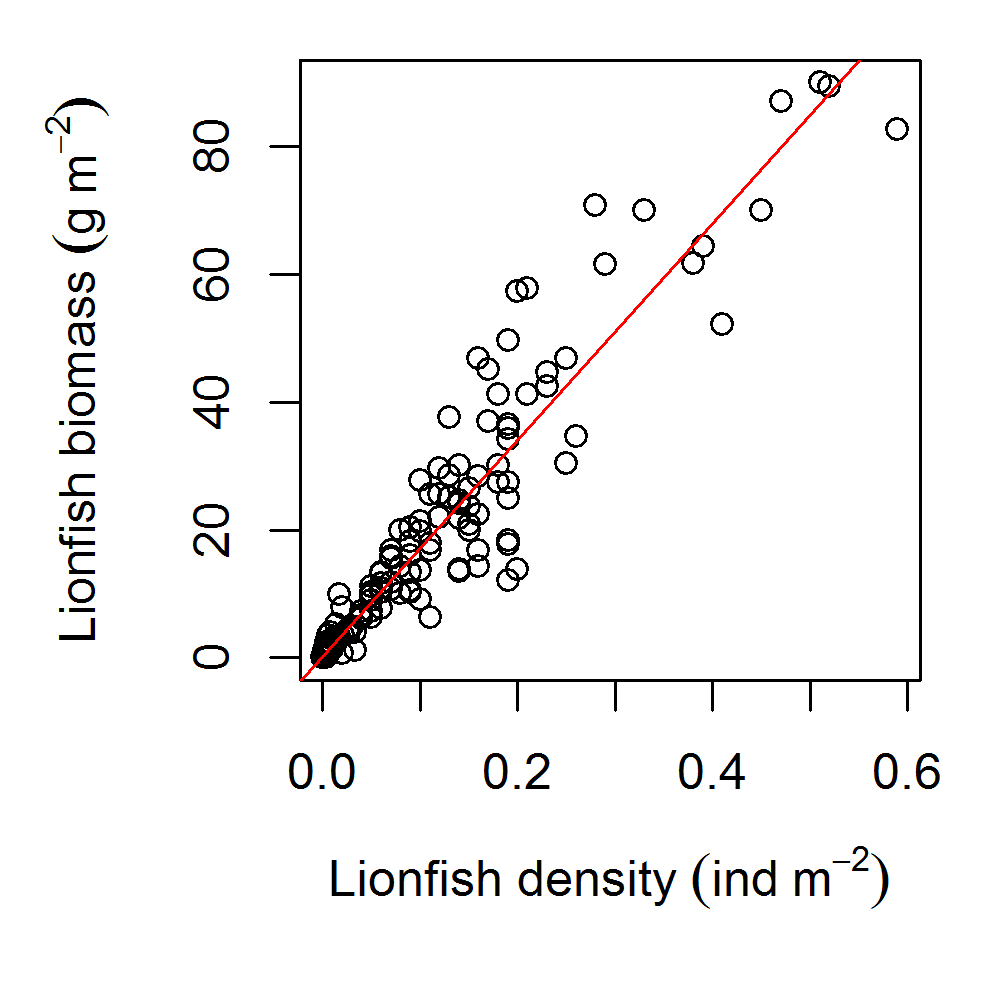

Supplement: Figure S1 — Relationship between lionfish density and biomass estimates. Each point represents a transect mean. The Pearson’s product-moment correlation between lionfish biomass and lionfish density was 0.95, p<0.01. (TIFF) [file pone.0068259.s001.tiff]

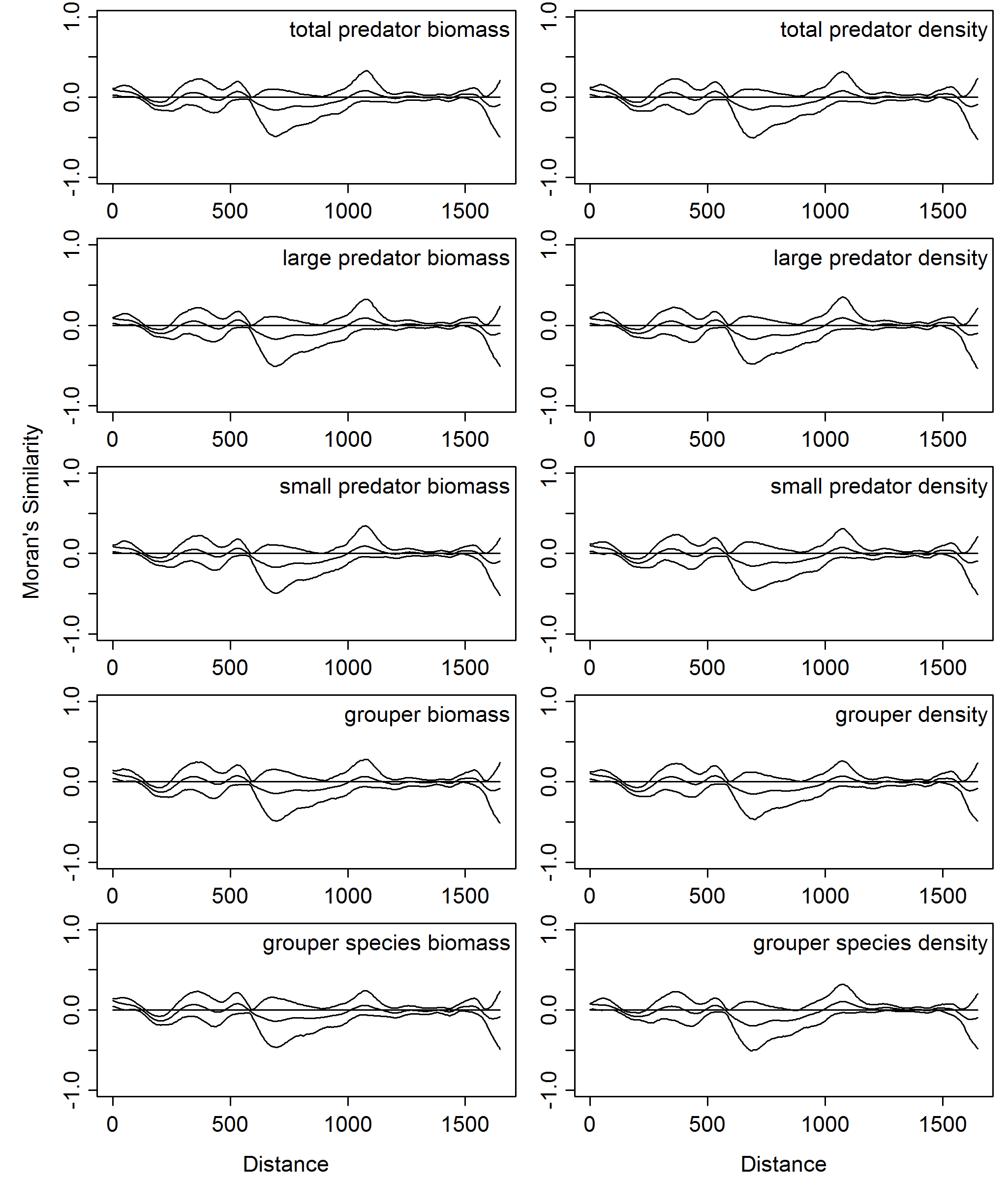

Supplement: Figure S2 — Spline correlograms, with 95% point wise bootstrap confidence intervals, of the Pearson residuals for each generalized liner mixed effects logistic regression model including all the explanatory variables fitted to the data. (TIFF) [file pone.0068259.s002.tiff]

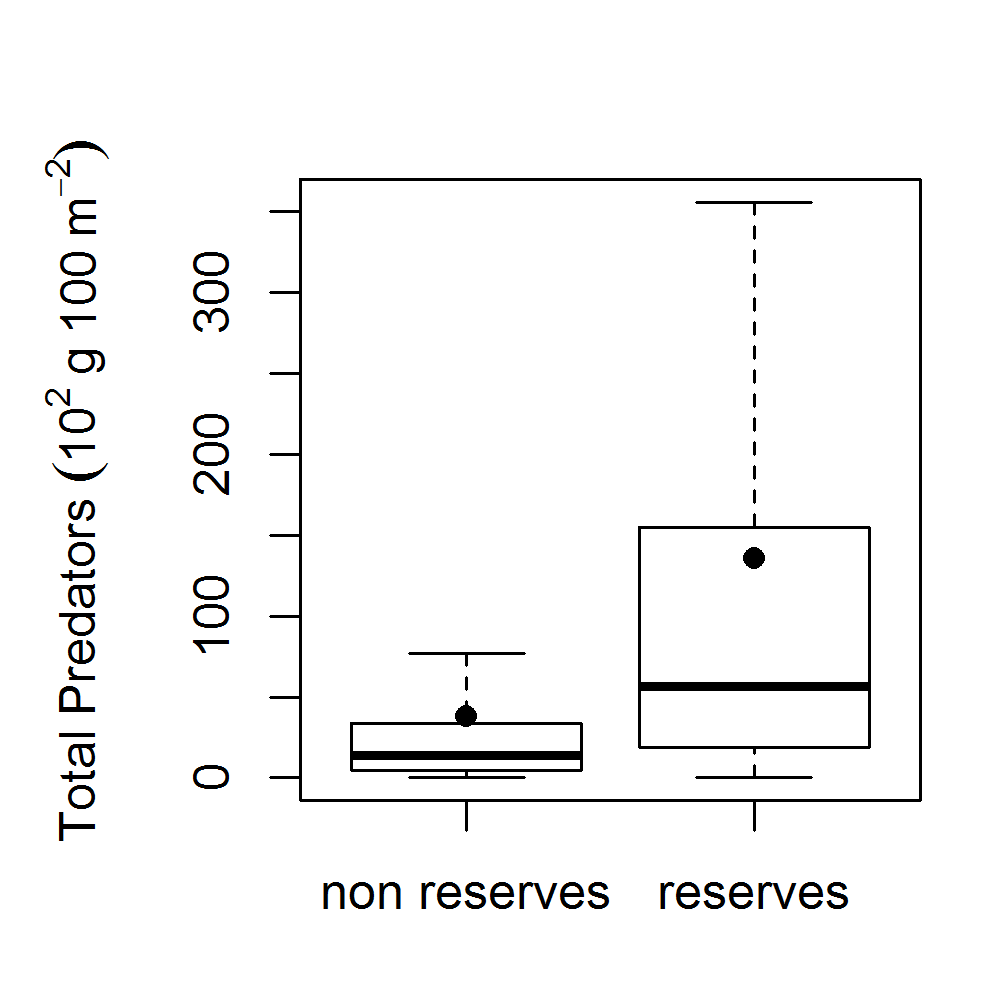

Supplement: Figure S3 — Total predator biomass on protected and unprotected Caribbean reefs. The biomass of native predatory fishes on 17 protected sites (no-take marine reserves) and on 55 unprotected or non-reserve reefs. Average predator biomass was significantly higher at sites inside marine reserves (135.4 g/m2) than in non-reserve sites (37.7 g/m2); t = −4.5933, p = 1.05e-05 (t-test). Boxplot shows the mean (black dot), median (black line) values. (TIFF) [file pone.0068259.s003.tiff]
