# Supplementary material for: Native Predators Do Not Influence Invasion Success of Pacific Lionfish on Caribbean Reefs
Source: PLoS One. 2013 Jul 11;8(7):e68259. doi: 10.1371/journal.pone.0068259 (PMC3708960; doi:10.1371/journal.pone.0068259)
Supplement: Table S1 — Survey locations across the Caribbean Study sites, site codes, regions, and protection level. Habitat type, S&G: Spur and Grove; Patch: Patch Reef. Protection level, NTZ: No-take zone; MPA: marine protected area; GUA: general used area. Permit, Yes: Permit Obtained (Permits for The Bahamas, Belize, and Mexico covered all sites); Not Req.: Permit was not required and therefore not obtained (For Cuba, only protected sites required a permit). (PDF) [file pone.0068259.s004.pdf]

Site name	Site code	Habitat type	Depth (m)	Date of survey	Latitude	Longitude	Protection level	MR year	Permit
Mesoamerican Barrier, Mexico									
Cancun	CU	S & G	12	7/02/12	21.02544	-86.7713	No protection		Yes
Cozumel North	CZN	S & G	10	7/03/12	20.47188	-86.9815	NTZ		Yes
Cozumel South	CZS	S & G	15	7/04/12	20.31961	-87.0266	NTZ		Yes
Chinchorro North	CN	S & G	15	7/05/12	18.74867	-87.3476	MPA		Yes
Chinchorro Central	CC	S & G	15	7/05/12	18.57457	-87.4198	MPA		Yes
Chinchorro South	CS	S & G	15	7/07/12	18.41008	-87.4169	MPA		Yes
Akumal	AK	S & G	15	7/08/12	20.42689	-87.2860	No protection		Yes
Mesoamerican Barrier, Belize									
Tackle Box	TB	S & G	12-15	5/21/12	17.91056	-87.95083	No protection		Yes
Hol Chan	HC	S & G	12-15	5/21/12	17.86343	-87.97238	NTZ	1987	Yes
Half Moon Caye	HM	S & G	12-15	5/25/12	17.20560	-87.54679	NTZ	1982	Yes
Calabash Caye	CA	S & G	12-15	5/25/12	17.26147	-87.81970	No protection		Yes
Middle Caye	MC	S & G	12-15	5/26/12	16.73703	-87.80536	NTZ	1993	Yes
South Middle Caye	SC	S & G	12-15	5/26/12	16.72875	-87.82867	NTZ	1993	Yes
Southwest Caye	ST	S & G	12-15	5/27/12	16.11247	-88.27107	No Protection		Yes
Pampion Caye	PO	S & G	12-15	5/27/12	16.37310	-88.08913	No Protection		Yes
Ranguana Caye	RA	S & G	12-15	5/29/12	16.28501	-88.15031	No Protection		Yes
Nicholas Caye	NI	S & G	12-15	5/28/12	16.11230	-88.25586	MPA/GUA	2003	Yes
South Water Caye	SW	S & G	12-15	5/30/12	16.81346	-88.07756	MPA/GUA	1996	Yes
Tobacco Caye	TO	S & G	12-15	5/30/12	16.91911	-88.04757	No protection		Yes
Alligator Caye	AL	S & G	12-15	5/31/12	17.19660	-88.05115	No protection		Yes
Bay of Pigs, Cuba									
Cueva Peces	CP	Slope	10-12	6/15/10	22.16627	-81.13827	No protection		Not Req.
Punta Perdiz	PZ	Slope	8-10	6/16/10	22.11003	-81.11626	No protection		Not Req.
Ebano	EB	Slope	10-12	6/17/10	22.07914	-81.07599	No protection		Not Req.
Bacunayagua, Cuba	BC	Slope	10-12	6/16/12	23.14653	-81.66664	No protection		Not Req.
Jardines de la Reina, Cuba									
Cueva Pulpo	CF	Slope	10-12	5/29/11	20.75266	-78.83634	MPA/Nat Park	1996	Yes
Five Sea	CF	Slope	10-12	5/30/11	20.76177	-78.85222	MPA/Nat Park	1996	Yes
Anclita	AN	Slope	10-12	5/31/11	20.78697	-78.94317	MPA/Nat Park	1996	Yes
El Peruano	EP	Slope	10-12	6/01/11	20.84411	-79.02166	MPA/Nat Park	1996	Yes
Pipin	PP	S & G	12-15	6/02/11	20.82586	-78.98026	MPA/Nat Park	1996	Yes
Abaco, Bahamas									
Rocky Point	RP	Slope	10-12	7/05/11	25.99661	-77.40092	Remote		Yes
Man o' War	MW	S & G	10-12	7/09/11	26.62122	-77.00550	No protection		Yes
Guana Cay	GC	S & G	10-12	7/11/11	26.70967	-77.15408	No protection		Yes
Little Harbor	LH	S & G	10-12	7/12/11	26.32390	-76.99160	No protection		Yes
Pelican Cay	PC	Slope	8-10	7/19/11	26.39783	-76.98850	NTZ	1972	Yes
Fowls Cay	FC	Slope	8-10	7/20/11	26.63717	-77.03848	NTZ	2009	Yes
New Providence, Bahamas									
Bond Wrecks	BW	S & G	12	6/10/10	25.00850	-77.55717	No protection		Yes
David Tucker	DT	S & G	14	6/04/10	25.00450	-77.55383	No protection		Yes
DC3 Wall	DW	S & G	14	6/10/10	25.00616	-77.55200	No protection		Yes
Mike's Reef	MK	S & G	13	6/12/10	24.97066	-77.53417	No protection		Yes
Pumpkin Patch	PK	S & G	12	7/06/10	24.97443	-77.53517	No protection		Yes
RSP Group	RS	S & G	15	6/08/10	24.91061	-77.52632	No protection		Yes
Willaurie	WI	S & G	15	6/24/10	25.01535	-77.56977	No protection		Yes
Eleuthera, Bahamas									

100	100	Patch	3	11/28/09	24.82791	-76.24382	No protection	Yes
101	101	Patch	3	11/28/09	24.82643	-76.24825	No protection	Yes
102	102	Patch	3	11/28/09	24.82422	-76.25333	No protection	Yes
104	104	Patch	3	11/26/09	24.82758	-76.26625	No protection	Yes
106	106	Patch	3	11/28/09	24.81817	-76.24953	No protection	Yes
108	108	Patch	3	11/29/09	24.81740	-76.24415	No protection	Yes
112	112	Patch	3	11/28/09	24.82102	-76.25728	No protection	Yes
55	55	Patch	3	11/29/09	24.84454	-76.26042	No protection	Yes
70	70	Patch	3	11/25/09	24.95057	-76.28712	No protection	Yes
71	71	Patch	3	11/25/09	24.83282	-76.27265	No protection	Yes
72	72	Patch	3	11/24/09	24.86077	-76.25942	No protection	Yes
73	73	Patch	3	11/24/09	24.85883	-76.25428	No protection	Yes
74	74	Patch	3	11/24/09	24.85712	-76.25735	No protection	Yes
75	75	Patch	3	11/29/09	24.85253	-76.25510	No protection	Yes
76	76	Patch	3	11/23/09	24.85208	-76.25828	No protection	Yes
77	77	Patch	3	11/23/09	24.85302	-76.24918	No protection	Yes
78	78	Patch	3	11/23/09	24.85275	-76.24708	No protection	Yes
79	79	Patch	3	11/25/09	24.84423	-76.24725	No protection	Yes
80	80	Patch	3	12/25/09	24.84107	-76.24452	No protection	Yes
84	84	Patch	3	11/26/09	24.83398	-76.24285	No protection	Yes
89	89	Patch	3	11/24/09	24.84838	-76.24215	No protection	Yes
90	90	Patch	3	11/25/09	24.84635	-76.25417	No protection	Yes
91	91	Patch	3	11/25/09	24.84968	-76.25112	No protection	Yes
93	93	Patch	3	11/26/09	24.83670	-76.26080	No protection	Yes
94	94	Patch	3	11/26/09	24.81940	-76.26097	No protection	Yes
