# Supplementary material for: Native Predators Do Not Influence Invasion Success of Pacific Lionfish on Caribbean Reefs
Source: PLoS One. 2013 Jul 11;8(7):e68259. doi: 10.1371/journal.pone.0068259 (PMC3708960; doi:10.1371/journal.pone.0068259)
Supplement: Table S2 — Reef fish predator species used in the study. Taxonomic information, food guild and trophic groups of the predator species used in the analysis. Guild and trophic information was obtained from Fish-Base [16]. (PDF) [file pone.0068259.s005.pdf]

Family	Common Name	Species Name	Food Guild	Trophic	Predator/Competitor
Aulostomidae	Trumpetfish	*Aulostomus maculatus*	Piscivore	4.26	Competitor
Belonidae	Houndfish	*Tylosurus crocodilus*	Piscivore	4.47	Competitor
Bothidae	Peacock Flounder	*Bothus lunatus*	Piscivore	4.45	Competitor
Carangidae	Bar Jack	*Carangoides ruber*	Piscivore	4.39	Competitor
Carangidae	Yellow Jack	*Caranx bartholomaei*	Piscivore	4.50	Predator/Competitor
Carangidae	Blue Runner	*Caranx crysos*	Piscivore	4.40	Predator/Competitor
Carangidae	Crevalle Jack	*Caranx hippos*	Piscivore	3.50	Predator/Competitor
Carangidae	Horse Eye Jack	*Caranx latus*	Piscivore	4.36	Predator/Competitor
Carangidae	Rainbow Runner	*Elagatis bipinnulata*	Piscivore	3.59	Predator/Competitor
Carangidae	Greater Amberjack	*Seriola dumerili*	Apex predator	4.50	Predator
Carangidae	Almaco Jack	*Seriola rivoliana*	Apex predator	4.50	Predator
Carangidae	Palometa	*Trachinotus goodei*	Piscivore	4.32	Predator/Competitor
Carcharhinidae	Silky Shark	*Carcharhinus falsiformis*	Apex predator	4.50	Predator
Carcharhinidae	Blacktip Shark	*Carcharhinus limbatus*	Apex predator	4.24	Predator
Carcharhinidae	Reef Shark	*Carcharhinus perezi*	Apex predator	4.50	Predator
Carcharhinidae	Lemon shark	*Negaprion brevirostris*	Apex predator	4.37	Predator
Centropomidae	Common Snook	*Centropomus undecimalis*	Piscivore	4.41	Predator/Competitor
Dasyatidae	Southern Stingray	*Dasyatis americana*	Piscivore	3.51	Competitor
Elopidae	Tarpon	*Megalops atlanticus*	Apex predator	4.50	Predator/Competitor
Haemulidae	Sailors Choice	*Haemulon parra*	Piscivore	3.47	Competitor
Haemulidae	White Grunt	*Haemulon plumieri*	Piscivore	3.60	Competitor
Lutjanidae	Mutton Snapper	*Lutjanus analis*	Piscivore	3.86	Predator/Competitor
Lutjanidae	Schoolmaster	*Lutjanus apodus*	Piscivore	4.17	Predator/Competitor
Lutjanidae	Cubera Snapper	*Lutjanus cyanopterus*	Apex predator	4.22	Predator/Competitor
Lutjanidae	Gray Snapper	*Lutjanus griseus*	Piscivore	4.25	Predator/Competitor
Lutjanidae	Dog Snapper	*Lutjanus jocu*	Piscivore	4.30	Predator/Competitor
Lutjanidae	Mahogany Snapper	*Lutjanus mahogoni*	Piscivore	4.36	Competitor
Lutjanidae	Lane Snapper	*Lutjanus synagris*	Piscivore	3.82	Competitor
Lutjanidae	Yellowtail Snapper	*Ocyurus chrysurus*	Piscivore	3.99	Competitor
Muraenidae	Green Moray	*Gymnothorax funebris*	Apex predator	4.01	Predator/Competitor
Rhincodontidae	Nurse Shark	*Ginglymostoma cirratum*	Apex predator	3.83	Predator/Competitor
Scombridae	King Mackerel	*Scomberomorus caballa*	Piscivore	4.50	Predator/Competitor
Scombridae	Cero	*Scomberomorus regalis*	Apex predator	4.48	Predator/Competitor
Scorpinadae	Lionfish	*Pterois volitans*	Piscivore	4.45	
Serranidae	Graysby	*Cephalopholis cruentata*	Piscivore	4.23	Predator/Competitor
Serranidae	Coney	*Cephalopholis fulva*	Piscivore	4.05	Predator/Competitor
Serranidae	Rock Hind	*Epinephelus adscensionis*	Piscivore	3.51	Predator/Competitor
Serranidae	Red Hind	*Epinephelus guttatus*	Piscivore	3.88	Predator/Competitor
Serranidae	Jewfish	*Epinephelus itajara*	Piscivore	4.09	Predator/Competitor
Serranidae	Red Grouper	*Epinephelus morio*	Piscivore	3.49	Predator/Competitor
Serranidae	Nassau Grouper	*Epinephelus striatus*	Piscivore	4.06	Predator/Competitor
Serranidae	Shy Hamlet	*Hypoplectrus guttavarius*	Piscivore	3.81	Competitor
Serranidae	Indigo Hamlet	*Hypoplectrus indigo*	Piscivore	3.85	Competitor
Serranidae	Black Hamlet	*Hypoplectrus nigricans*	Piscivore	3.85	Competitor
Serranidae	Barred Hamlet	*Hypoplectrus puella*	Piscivore	3.65	Competitor
Serranidae	Butter Hamlet	*Hypoplectrus unicolor*	Piscivore	3.65	Competitor
Serranidae	Black Grouper	*Mycteroperca bonaci*	Apex predator	4.50	Predator/Competitor
Serranidae	Yellowmouth	*Mycteroperca*	Apex predator	4.50	Predator/Competitor
Serranidae	Tiger Grouper	*Mycteroperca tigris*	Apex predator	4.50	Predator/Competitor
Serranidae	Yellowfin Grouper	*Mycteroperca venenosa*	Apex predator	4.49	Predator/Competitor
Serranidae	Greater Soapfish	*Rypticus saponaceus*	Piscivore	4.06	Predator/Competitor
Sphyraenidade	Great Barracuda	*Sphyraena barracuda*	Apex predator	4.50	Predator/Competitor
Sphyraenidade	Southern Sennet	*Sphyraena picudilla*	Piscivore	4.50	Predator/Competitor
