# Supplementary material for: Native Predators Do Not Influence Invasion Success of Pacific Lionfish on Caribbean Reefs
Source: PLoS One. 2013 Jul 11;8(7):e68259. doi: 10.1371/journal.pone.0068259 (PMC3708960; doi:10.1371/journal.pone.0068259)
Supplement: Text S1 — Field survey permit information. (DOCX) [file pone.0068259.s006.docx]

**Text S1. Field survey permit information.**

**Bahamas**; Department of Marine Resources, Ministry of Agriculture and Marine Resources. Permit MAF/FIS/17. Director Roland Albury.

**Cuba**: Centro de Control y Inspección Ambiental, via Fabian Pina; Mexico: Dirección General de Ordenamiento Pesquero y Acuicola de la Comisión Nacional de Acuicultura y Pesca (CONAPESCA) de la Secretaría de Agricultura, Ganaderia, Desarrollo Rural, Pesca y Alimentación (SAGARPA). Permiso DAPA/2/06504/110612/1608. Director General. Lic. Aldo Gerardo Padilla Pestaño.

**Belize**: Belize Fisheries Department. Permit # 000028-11. Fisheries Administrator Beverly Wade].

No specific permission was required for the remaining sites because these locations were public or touristic dive sites where access was not restricted and the correspondent national agency did not require a permit.
